# Theories of Postdigital Heterogeneity: Implications for Research on Education and Datafication

**DOI:** 10.1007/s42438-021-00232-w

**Published:** 2021-05-15

**Authors:** Felicitas Macgilchrist

**Affiliations:** 1 Media Transformation, Georg Eckert Institute for International Textbook Studies, Braunschweig, Germany; 2 Institute for Educational Science, University of Goettingen, Goettingen, Germany

**Keywords:** Data assemblages, Ethnography, Infrastructure, Noise, Postdigital practice, Platformization

Postdigital theory assumes that the digital is no longer new, and disruption now mundane. It gets us beyond binaries such as online/offline, virtual/real, old/new media, digital/analogue, technical/natural, biological/informational (Cramer 2014; Jandrić et al. 2018).^1^ Rather than being shiny and new, we can, suggests Jörissen, ‘think of the digital as mycelium: the actual organism consists of the large invisible, interconnected, subterranean linkages’. What we think of as a mushroom is ‘only the mycelium’s fruiting body; a secondary manifestation’ (Jörissen 2017).^2^ With this metaphor, when we look at hardware, software, algorithms, data, etc., we are only looking at secondary manifestations. Like mycelia, the digital is more than the fruiting body; digitality is deeply woven throughout the contemporary world, and we thus live in a postdigital condition.

At the same time, the postdigital condition is one of ‘imperial and industrial ruin’ (Tsing et al. 2021). Precarity threads through many lives, whether due to personal economic circumstances or with a gaze to the future of the planet. The idea that technological progress is inevitably social progress has, for many observers, crumbled. One prominent reaction to this, however, is concern that humans are losing our autonomy and freedom as we face ‘a new global architecture of behaviour modification’ (Zuboff 2019) or today’s ‘emerging grid of surveillance and data extraction’ which is on ‘a truly global scale’ (Couldry and Mejias 2019).

I have been looking for concepts to help analyse education and datafication within this postdigital/precarious condition but outwith these universalising humanist concepts. I turned to theories of heterogeneity, i.e. the onto-epistemological assumption that the world is messy and that scholarship should thus engage with this messiness rather than try to smooth it out into generalisations and universals. These theories do not, I should add, suggest that the world was previously made of separate spheres that have become interwoven, but that there were never ontologically separate spheres in the first place (see also Coeckelbergh 2020). But given that the datafication and platformisation of education are interesting precisely because they seem like global phenomena (see Decuypere et al. 2021; Jarke and Breiter 2019), theories of heterogeneity may offer one way to follow up on Milan and Treré’s (2019) important injunction to ‘mov[e] past the “universalism” associated with our interpretation of datafication’.

Mess is one concept, polyphony another, practices are always complex. Fungi and rhizomes have proven helpful, as have feral ecologies and compost. Hybrid, interwoven and interconnected are problematic: they presuppose that elements first have a separate identity and then come together. Intra-active, intra-woven or intra-connected may work better for my purposes: they operate with the different presupposition that elements are constituted in, not before, relations. Sympoiesis (‘making-with’) seems exciting, flagging a ‘with’ rather than the ‘self’ of autopoiesis. The friction of tyres on roads or the noisy hum of daily life could be good to think with.

From the broad array, I shall try out three concepts in a little more detail to think about the postdigital: postdigital assemblages, postdigital practices and postdigital noise. These can be seen as alternatives or as complementary concepts. Although they overlap a good deal, each opens up slightly different priorities. The conclusion reflects on some implications for educational research.

## Postdigital Assemblages

Tsing (2015) draws on ecologists and on Deleuze’s *agencement* for her notion of assemblage, adding ‘polyphonic’ to emphasise that the kind of assemblage she finds helpful is about knots, not networks, about human and nonhuman ‘ways of being’ as ‘emergent effects of encounters’, rather than community influence. Polyphonic assemblages are inspired by music such as the madrigal or fugue; when we listen, we can ‘pick out separate, simultaneous melodies’ *and* ‘the moments of harmony and dissonance they created together’ (Tsing 2015: 24). We hear the multiple temporal rhythms of the assemblage. Pennycook (2018) and Gourlay and colleagues (2021) write of semiotic assemblages to emphasise the gathering of human and nonhuman actors. Haraway (2017) suggests ‘symbiotic assemblages’, which are ‘like knots of diverse intra-active relatings in dynamic complex systems’, i.e. quite explicitly *not* made up of pre-existing bounded units, like genes or cells or organisms (Haraway 2017: M26). Her assemblages are about attachments and diverse relationalities; they aim to overcome biology’s ‘methodological individualism’.

These kinds of assemblages resonate with the notion of ‘data assemblages’ as heterogeneous ensembles that evolve and mutate over time (Kitchin and Lauriault 2018). Tsing, however, emphasises the constitutive indeterminacy of assemblages and the spaces of encounter in which ways of being come together. Haraway criticises biology’s assumption that the individual units exist before the assemblage, rather than coming into being within it. Williamson and Eynon (2020) tease out historical assemblage of threads around AI in education. In these ways, these writers gently interrupt the classificatory practices of scholars that divide assemblages into lists of elements or components or stages or processes (see also Piattoeva and Saari 2020).

Adding postdigital as an adjective, ‘postdigital assemblages’ would flag the ways data assemblages are held together, rather than describing their elements. Which kinds of political economy do postdigital assemblages drag inside them? What knots are visible that can be unravelled to show what is coming into being and how things are holding together in this particular classroom on this particular day? Which trajectories combine? What relatings can we see? Which multifarious little details can be told about big concepts such as digital transformation, big data or datafication? What histories are wrapped up in the practices?

Working with the concept of postdigital assemblage, I imagine starting with an interview with a teacher who reports on her students using specific software while working on specific tasks. I unravel the knots towards the data dashboard, the software developers, the software development kit and integrated development environment, the privacy settings, the teachers’ notes, the sources of funding, the teacher room conflicts over which technology to use for what purposes, the policy on digital learning, the infrastructuring of software and more. But when I write that sentence, I notice I have divided the assemblage into elements after all. Thinking with the postdigital assemblage reminds me to avoid making this list systematic, but to think instead of how to unravel and ravel up—to recombine—them into a complex, heterogeneous and historicised story anchored in that teacher’s account.

## Postdigital Practices

Influential in social science, practice theory has led to rich research findings. Again, however, in overview articles or introductory books, authors tend to identify elements or components that make up practices, sometimes dividing up ‘social practices’ from, e.g. ‘discursive practices’, which can lead to headaches over where, precisely, to draw the boundaries. I want, therefore, to draw here on Abu-Lughod’s short, heterogeneous account of practices. She prioritises practice theory which is ‘built around problems of contradiction, misunderstanding and misrecognition, and favours strategies, interests and improvisations’ (Abu-Lughod 1991: 472). Her argument at the time of writing was against generalisations and ‘the more static and homogenizing cultural tropes of rules, models and texts’, but it works equally well against today’s generalisations and abstractions about datafication.

Scholars referring to ‘postdigital practices’ have also oriented to heterogeneity. Fleischer (2015), for instance, aims to complexify a narrow understanding of postdigital aesthetics by analysing how music practices (from a revitalised cassette culture to dubstep) subvert the contemporary global processes of commodification that are underway as we increasingly have unlimited access to music. Götz (2019) analyses how Buttendorf’s piece ‘#HotPhones—high-tech selfcare’, a participatory relaxation workshop for and with smartphones and their users, positions itself between the binary of identifying with today’s platform/surveillance/etc. capitalism or rejecting it. The piece complexifies self-care and notions of agency and participation, by offering alternative practices that use connectivity to escape connectivity.

Practices are also about the quotidian, the apparently banal and the habitual (Chun 2016). For Ryberg, ‘the postdigital is about dragging digitalisation and the digital—kicking and screaming—down from its discursive celestial, ethereal home and into the mud’ (Jandrić et al. 2019: 166). Practices in the mud include ‘managing a class of 7-year-olds working on tablets (half of them not charged and the other half with links to dubious sites)’.

Working with the concept of postdigital practices, I imagine starting with an observation of a specific moment, perhaps a student I saw pulling out his smartphone, plugging in his earphones and selecting some music before he grudgingly swept the classroom floor at the end of a school day. What music is it? Does he listen to automatically generated recommendations? How do the earphones connect him to somewhere else, disconnect him from the chatter in the classroom and connect his movements to the floor? How is his participation in the global commodification of music through streaming platforms simultaneously a kind of self-care and care for his classroom community for whom he is cleaning? By noticing this moment, these are only some of the aspects that I would combine into a more complex story that tries to understand (postdigital) schooling through these practices.

## Postdigital Noise

Noise is different from contradiction, misunderstanding and misrecognition. I draw here on the notion of noise in data science. In this context, noise refers to corrupt, incorrect or meaningless data. Noisy data are strange, they don’t fit expectations. Data scientists acknowledge that most complex data sets include noise. Noise is not a glitch; it is a constant feature of real-life data sets. We hear noise when we notice that data are ‘broken’ (Pink et al. 2018). Where data science would often like to sooth or remove the noise, theories of heterogeneity want to listen more closely for insights into how datafication is enacted in today’s capitalist ruins.

In her ethnography of global connection, Tsing sketches two approaches to studying capitalism. In one, scholars prioritise the universalising quality of capitalism and make particularities into instances of the universal. In the other, scholars identify specificity and trace place-based struggles against exploitation. These two positions resonate strongly with contemporary research on datafication: in one, surveillance capitalism, data colonialism and similar analyses and, in the other, data feminism, data justice and data activism.^3^

As Tsing writes, the ‘contribution of each of these works is stunning; yet placed in conversation they seem to block each other’ (Tsing 2005: 4), for instance, when data activism is reduced to an insufficient, partial and temporary tactic (Couldry and Mejias 2019: 194f; for an alternative view, see Benjamin 2019; Dencik et al. 2019; D’Ignazio and Klein 2020; Gutiérrez 2018). By operating at the intersection of the two approaches, Tsing focuses on ‘zones of awkward engagement’ (Tsing 2005: xi), which I read here as spaces in which the noise becomes discernible. She identifies how the messiness of capitalist forms arises when universals are put to action in specific encounters. ‘Odd connections’ are the result of this kind of research, rather than ‘seamless generalizations’ (2005: xi), and universals are seen as ‘sticky engagements’ in a heterogeneous world (2005: 6).

This stickiness points to the noisy sociomateriality of digital technology which is emphasised in, among other work, studies on the postdigital. Despite the marketing department’s orientation to smooth, harmonious customer engagement, the digital includes noise; it needs matter, bodies, labour, etc. to mine (noisily) for minerals, to build machines in (loud) workshops (Crawford and Joler 2018; Gorur and Dey 2021; Knox 2019). Nor do digital technologies always operate softly, as many of us experienced during the Covid-19 pandemic. More specifically, Scourti’s (2015) ‘Think You Know Me’, which creates poetry through the predictive text of her smartphone, foregrounds the noise (i.e. corrupt, incorrect and meaningless data) in today’s datafied regimes of prediction (Bishop et al 2015: 22). Noise is more than an occasional clash; it is constitutive of (global) connection.

Working with this concept of postdigital noise, I imagine starting with a document on data infrastructure for schools in Germany. I see that universals and standardisations are featured prominently (the goals are interoperability and expandability; connectivity with transnational systems; equity and participation for all). I would trace how the people working with the material infrastructures engage with these universal rhetorics, how they deal with the practical dilemmas they face, which workarounds they find for when the noise becomes palpable and things don’t work smoothly (see Whitman 2020). The document emphasises, as do many in Germany, the primacy of the pedagogical (in opposition to a primacy of the technical that was diagnosed in the past). But assuming that educational practices are constituted in and with the technology, neither before nor after, I would explore how this appeal to the teacher’s autonomy and freedom unfolds in specific, noisy, ruffled educational encounters. Perhaps in schools that are trialling infrastructures provided by global corporations.

Thinking with postdigital noise suggests that precisely the ‘unpredictability’ of (fast) policy (Peck and Theodore 2015: 32) is the most intriguing part to explore. The goal, rather than starting with a critique of imperial data power and then identifying ways to escape this (and retain/regain our human dignity), is to acknowledge that we live in the midst of planetary ruins and nevertheless ‘maintain enough curiosity to notice the strange and wonderful as well as the terrible and terrifying’ (Swanson et al. 2017: M7; see also Tsing et al. 2021).

## Concluding Thoughts

Universals can be seductive. In this short piece, which is by no means thorough or complete, I have suggested instead that theories of heterogeneity take us to exciting analytical places. Postdigital assemblages, practices and noise divert our attention away from the dynamics of scale in the (global) expansion and standardisation of data infrastructures and policies. Instead, they alert us to what is going on in the mycelia, the mud or the lines of flight; they help us notice the multiple, layered, intra-woven rhythms of world-making and decomposition today.

Some readers may wonder if the adjective ‘postdigital’ is really necessary. Perhaps not, but words do things. By flagging the attempt to overcome a troublesome binary that still dominates public and academic discussions of technology and education, this adjective opens up what Tsing (2015) has called arts of noticing. The prefix ‘post-’ also returns us to the mycelia’s underground work. As Sinclair and Hayes note, thinking ‘with’ (com-) the ‘post-’ of postdigital takes us to com-post—often enriched by fungal mycelia—that can be ‘fertile ground’ (Sinclair and Hayes 2018: 129) for theory and empirical research.

Three implications for research conclude this piece. First, the economy of knowledge, including citational practices, mean that universalising titles are picked up and circulated more easily than writings which explore, e.g. the complex noise that make it ‘impossible for the bottom line to be one single statement’ (Haraway 2000: 105). Theorists of heterogeneity, such as those cited in this article, remind us to reflect on who we think with when we do our research.

Second, patchwork ethnographies (Tsing) or ethnographies of the particular (Abu-Lughod) are only one set of methodologies emerging from these theories that will be important for future studies of datafication and education. This is not only about following policy or educational technologies into classrooms but also about what we notice when we look into classrooms. The broader implication for research is to attend to the heterogeneity. I suggested some ideas above which draw on interviews, observations and analysis of documents, software and infrastructure.

Third, none of the three concepts offers the redemption, solutions or ways out of our contemporary conundrum that educational researchers are often expected to provide. By analysing heterogeneity, however, we might avoid the seductions of abstractions about the digital and find new ways of noticing and weaving together the terrible, the terrifying, the strange and the wonderful of the postdigital.

## Footnotes

1 Other concepts (onlife, sociomaterial, sociotechnical, materialsemiotic, naturecultures) aim to overcome similar binaries.

2 Authorʼs translation from German.

3 For Tsing, social scientists tend towards universality and humanities scholars tend towards place-based specificity. Although the two kinds of research are still prevalent, I am not sure this disciplinary split holds up (any longer). There are also sufficient scholars (too many to cite here) who step outside this binary and analyse the complexities and fractures of datafication. My main argument here is against those studies which Viljoen (2021) describes as ‘dignitarian’ approaches, i.e. humanist approaches that fear a (universal) loss of human dignity in the face of commodified data extraction.
